# Physiological and Behavioral Effects of SiO_2_ Nanoparticle Ingestion on *Daphnia magna*

**DOI:** 10.3390/mi12091105

**Published:** 2021-09-14

**Authors:** Youngsam Kim, Afshin Samadi, Eun Heui Gwag, Jayoung Park, Minjeong Kwak, Jihoon Park, Tae Geol Lee, Young Jun Kim

**Affiliations:** 1 Environmental Safety Group, KIST Europe Forschungsgesellschaft mbH, 66123 Saarbrücken, Germany; youngsam.kim@kist-europe.de (Y.K.); afshin.samadi@kist-europe.de (A.S.); eunheui.gwag@kist-europe.de (E.H.G.); pjy0404@gmail.com (J.P.); 2 Division of Energy & Environment Technology, University of Science & Technology, Daejeon 34113, Korea; 3 Center for Nano-Bio Measurement, Division of Industrial Metrology, Korea Research Institute of Standards and Science, 267 Gajeong-ro, Yuseong-gu, Daejeon 34113, Korea; kwakmj@kriss.re.kr (M.K.); tglee@kriss.re.kr (T.G.L.); 4 Accident Response Coordination Division, National Institute of Chemical Safety, Ministry of Environment, 11 Osongsaengmyeong-ro, Heungdeok-gu, Cheongju 28164, Korea; ichkann@korea.kr

**Keywords:** *D. magna*, silicon dioxide nanoparticles, swimming performance, heart rate

## Abstract

The increasingly widespread use of engineered nanoparticles in medical, industrial, and food applications has raised concerns regarding their potential toxicity to humans and the environment. Silicon dioxide nanoparticles (SiO_2_ NPs), which have relatively low direct toxicity, have been increasingly applied in both consumer products and biomedical applications, leading to significantly higher exposure for humans and the environment. We carried out a toxicity assessment of SiO_2_ NPs using the common water flea *D. magna* by focusing on physiological and behavioral indicators such as heart rate, swimming performance, and growth. Exposure to SiO_2_ NPs did not produce acute or chronic toxicity at limited concentrations (<100 μg/mL), but did have statistically significant negative effects on heart rate, swimming distance, and body size. The use of fluorescein isothiocyanate in a silica matrix allowed the tracing and visualization of clear SiO_2_ NP accumulation in *D. magna*, which was confirmed by ICP-MS. Although exposure to SiO_2_ NPs seemed to affect cardiac and swimming performance, such end-point experiments may be insufficient to fully understand the toxicity of these nanoparticles. However, the physiological and behavioral changes shown here suggest potential adverse effects on the aquatic environment by substances previously considered nontoxic.

## 1. Introduction

Engineered nanoparticles have been used in a broad range of fields due to their exceptional advantages compared to conventional bulk materials. Their diverse physicochemical, mechanical, and biocompatible properties have led to a tremendous increase in their production and usage [1,2,3]. Silicon dioxide nanostructures are among the most commonly produced nanoparticles globally due to their benefits, low toxicity and cost, and ease of production. Globally, ~1.5 million t of silicon nanoparticles (SiO_2_ NPs) have been produced, making them one of the most produced nanomaterials since 2013 [4,5,6]. These materials are widely used in consumer products such as ceramics, glass, medicine, and cosmetics, but have been extended to biomedical applications given their stabilization properties and low toxicity [7,8]. Given that the possibility of exposure to humans and leakage into the environment has increased significantly, their potential for environmental damage or adverse effects should also be considered with respect to their interactions with living organisms. Although the potential toxic effects on human health have been aggressively explored by in vitro and in vivo studies [9,10,11], toxicological research on environmental effects is lacking, despite growing exposure to various ecosystems.

In the last two decades, researchers have consistently assessed the aquatic toxicity of SiO_2_ NPs using key parameters such as size, function, and morphology [12], which are considered crucial factors because of their potential roles in toxicity. Surface modification techniques, such as functionalized and coated nanoparticles, give rise to different properties through covalent bonds and ionic coordination, resulting in alterations in toxicity [13,14,15]. Particle size is also an important factor in understanding toxicity because the surface-to-volume ratio and surface reactivity increase as particle size decreases. Previous studies have demonstrated that a decrease in size induces greater toxicity [16,17]. A wide range of particle sizes have been found absorbed into the gastrointestinal system of *D. magna*, a water flea widely used for aquatic toxicology tests, while nanoparticles have been observed in almost all tissues of this species [18]. In particular, similar size-dependent effects on immobilization and mortality have also been reported for titanium-, aluminum-, copper-, and other metal-based nanoparticles [19,20]. From a morphological perspective, shape-based toxicity assessments have received less attention than those of other key parameters, and these have tended to focus on amorphous forms given their usage and toxicity, such that pristine SiO_2_ NPs (e.g., spherical, nonporous, and unfunctionalized) have received less research focus than functionalized amorphous nanoparticles [21,22]. However, spherical SiO_2_ NPs should not be overlooked in toxicity assessments, as new possibilities for their use and production are being reported in a variety of fields [23,24].

As a test subject for biological laboratory studies, *D. magna* has many benefits, including high sensitivity to the surrounding environment, economical handling, and short lifespan for monitoring from neonates to adults [25]. *D. magna* is a key species with regard to its geographical distribution and role in the food webs of freshwater ecosystems, which elevates its importance as a key species in ecotoxicology [26]. Additionally, its transparency allows the observation of optical parameters efficiently via noninvasive methods [27]. Given these advantages, acute and reproductive toxicity tests for the use of *D. magna* as an environmental testing organism are well-established by the Organisation for Economic Co-operation and Development (OECD) and International Organization for Standardization (ISO) [28]. Furthermore, behavioral and physiological changes in *D. magna* have been reported with respect to the toxicity of various substances under sublethal concentrations [29,30,31,32]. For example, swimming performance and heart rate have been used to test for the effects of drugs, chemicals, and particles. 

In this study, we observed physiological, behavioral, and chronological changes in *D. magna* to better understand the effects of SiO_2_ NPs at below sublethal concentrations, and used tracing to determine the relationship between SiO_2_ NPs accumulation and changes in *D. magna*. To enable visualization, fluorescein 5 (6)-isothiocyanate- (FITC) molecules were incorporated into the SiO_2_ NPs.

## 2. Materials and Methods

### 2.1. D. magna Culture

*D. magna* from ephippia were purchased from Micro Biotests Inc. (Belgium) and hatched under a 16/8 h light/dark regime using 7000 lux light for 72 h. The population was then maintained at <15 per glass beaker containing 1.5 L of Elendt M4 medium at 20.0 ± 1.0 °C under the same light cycle in a climate incubator. *D. magna* were fed daily on 0.1 mg C per individual with *Chlorella vulgaris* (~1.5 × 10^8^ cells/mL) purchased from the Culture Collection of Algae at Cologne University (Germany); 0.5 μL/mL of yeast, cerophyll, and trout chow (YCT) was also supplied three times a week. The beakers and culture medium were replaced three times a week to ensure water quality, and new neonates were removed daily to prevent crowding. The pH and dissolved oxygen content of all culture and testing media were checked before replacement. The reliability of the test conditions was regularly checked using potassium dichromate (Sigma-Aldrich, St. Louis, MO, USA) as a reference substance.

### 2.2. Synthesis and Characterization of SiO_2_ NPs

The synthesis and mechanisms of dye-doped SiO_2_ NPs have been well-established [33]. FITC was chosen from among various conjugated fluorescent molecules for the advantages conveyed by its high quantum yield, biocompatibility, stability, and well-established synthesis with SiO_2_ NPs [33,34,35]. For synthesis, we used the same method as [34]. Scanning electron microscopy (SEM) and diffraction light scattering (DLS) were used to determine nanoparticle morphology and size. SEM images and hydrodynamic size were obtained using a HITACHI S-4800 and a Zetasizer Nano ZSP (Malvern), respectively. To determine the average particle size, 141 single particles were measured using ImageJ software. 

### 2.3. Immobilization Test

The acute immobilization tests followed the procedure described in OECD guideline 202 [36]. The third brood of 60 neonates (<24 h) was exposed to various concentrations of two SiO_2_ NP sizes (20 and 50 nm) on a log scale (0.01, 0.1, 1.0, 10, and 100 μg/mL), as well as those of the control series. Groups were divided into six replicates of five daphnids for each concentration of SiO_2_ NPs in ISO medium, which was added to six-well culture plates filled with 10 mL of media in a group for 48 h. During the acute tests, immobilization and mortality were checked at designated times (3, 6, 12, 24, and 48 h). The experiment was performed in duplicate.

### 2.4. Reproduction Test

The reproduction tests were based on OECD guideline 211 [37] and performed using static tests. *D. magna* was randomly pooled from the third brood. Eight daphnids were individually held at the two concentrations (1.0 and 10 μg/mL) as well as in the control series for 21 d. We set the control series under the same conditions, except for SiO_2_ NP exposure. The glass vessels were filled with 60 mL of solution in a 100 mL volume beaker. The SiO_2_ NPs were sonicated for 20 min before addition to the new media. Neonates were removed and counted daily. Mothers were fed daily with *C. vulgaris* (~1.5 × 10^8^ cells/mL, 0.1 mg C/*D. magna*) and thrice a week with YCT (0.5 μL/mL). The beakers and media were renewed three times per week. 

### 2.5. Growth Test

*D. magna length* was measured one per week using an optical microscope (Model CKX41, Olympus Inc., Tokyo, Japan). The magnification, selected based on individual size, ranged from 2–4×. For this purpose, *D. magna* were transferred onto glass slides with a few drops of the originating medium. Body length was determined from the center of the eye to the base of the apical spine by using ImageJ program. In addition, ten neonates from the third brood in each condition were randomly pooled to check their size, following the same procedure. 

### 2.6. Swimming Performance Monitoring

Swimming performance was monitored using a direct-read instrument (Model Zebrabox, View Point Life Science Inc., Lyon, France). This was measured after exposure to SiO_2_ NPs at various concentrations on the same log scale as above for 48 h. Daphnids were transferred into a 96-well plate separately and placed in the Zebrabox. To minimize the influence of sudden environmental changes, this plate was kept in the Zebrabox under complete darkness for 30 min before measurement. After stabilization, the swimming distance was recorded in tracking mode every minute for 80 min under darkness. A transparent background mode with a low detection threshold of 10 was used. Other threshold options for inactivity were 4 and 2. Real-time frequencies were analyzed using automated observation software (Zebralab-2, View Point Life Science Inc., Lyon, France). Swimming performance was calculated as distance per minute. Therefore, frames with no detected motion were excluded from the analysis.

### 2.7. Heart Rate Counting

Six neonates after the immobilization test were randomly selected for heartbeat counting under each exposure condition. Methylcellulose solution (4% *v*/*v*, Lot No. SLCC9072, Sigma-Aldrich Corp., St. Louis, MO, USA) was used to fix the neonates on a glass plate. Heart rate was recorded as a video file for 1 min using an optical microscope at 4× magnification (Model CKX41, Olympus Inc., Tokyo, Japan). The heart rate was counted manually using low-speed playback (×0.3) and repeated three times, with the mean values of each sample used for comparison.

### 2.8. Fluorescent Imaging

*D. magna* were rinsed three times and transferred to fresh medium for 5 min to avoid strong fluorescence from the stuck nanoparticles on the carapace. They were then rinsed three times again and transferred to a glass slide. Visualization was performed using a fluorescence microscope (ZEISS SteREO Discovery V8) with filter set 09 and ZEN 2.6 software. The filtration and camera exposure times were based on the control series to eliminate background autofluorescence. ImageJ software was used merge fluorescence images.

### 2.9. ICP-MS Analysis

To remove the influence of continuous adsorption due to the SiO_2_ NPs and the adhesive substances on the carapace surface, *D. magna* after observation for 24 d in fluorescent microscopy were placed in fresh media for another 3 d. Fresh media was renewed daily. Each group was thoroughly ground in 300 μL 5% HNO_3_ solution. The samples were then transferred to a glass container holding 1.5 mL 70% HNO_3_ (Sigma-Aldrich) and 5% H_2_O_2_ (Sigma-Aldrich, St. Louis, MO, USA) (1:3) mixture, and heated at 100 °C for 1 h for thorough sample digestion. The digested sample was diluted with 3 mL 5% HNO_3_. The amount of total silicon ions was measured by inductively coupled plasma mass spectroscopy (iCAP Q ICP-MS, Thermo Scientific) in triplicate. Before the measurement, the ICP-MS was washed with 5% HNO_3_ for 10 min. A calibration curve from 1–1000 μg/L was made with a silicon standard (FluKa, Munich, Germany). The measurement mode was STD, the detection dwell time was 10 ms, and the test was repeated 10 times for each sample. 

## 3. Results

### 3.1. Characterization of FITC-Adopted SiO_2_ NPs

**Figure 1:**
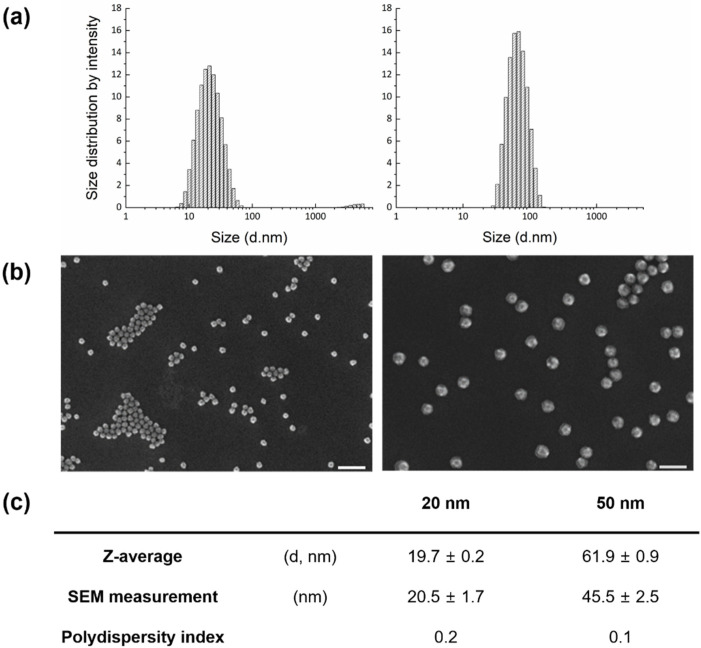
Characterization of synthesized silicon dioxide nanoparticles (SiO2 NPs): (**a**) Hydrodynamic sizes measured by diffraction light scattering (DLS); (**b**) representative scanning electron microscopy (SEM) images; and (**c**) particle information (n = 141 particles).

FITC-adopted SiO_2_ NPs were synthesized at 20 and 50 nm for visualization using a noninvasive method. To avoid functionalization on the SiO_2_ surface, a tetraethyl-orthosilicate (TEOS) precursor with covalent incorporation of FITC molecules was used to form a silica matrix. Both nanoparticle sizes were nearly spherical in shape, though slightly angular. The average sizes measured by DLS were slightly higher than those by SEM, but differences were negligible (Figure 1).

### 3.2. Acute and Chronic Effects of SiO_2_ NPs on D. magna Mortality and Reproduction

Acute toxicity tests were carried out to better understand this dynamic and to determine concentrations in a long-term exposure test at low concentrations. There was no significant mortality observed among the different groups, including the control series, for exposures up to 48 h (Figure S1). The chronic test was performed for two concentrations (1.0 and 10.0 μg/mL) based on the results of the short-term test, i.e., acute, swimming performance and heart rate. The mean number of neonates in the control series over 21 d was 78.7 ± 12.7, validated according to OECD guideline 211. There was no difference in reproductive output between the experimental and control groups, demonstrating that SiO_2_ NPs did not affect reproductive outcomes under the applied conditions (Figure S2).

### 3.3. Effects of SiO_2_ NPs on D. magna Swimming Performance and Heart Rate

**Figure 2:**
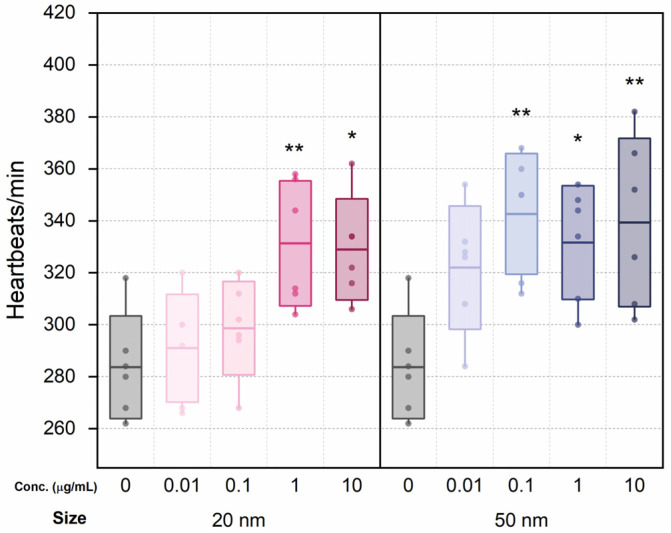
Heart rate changes following treatment with 20 and 50 nm SiO_2_ NPs after 48 h exposure. Boxes express standard deviations; central line indicates the mean. * and ** indicate *p* < 0.05 and *p* < 0.01 relative to the control group, respectively.

Six individuals were randomly selected from each group for heart rate testing. Although the heart rate did not show significant changes at low concentrations (0.01 and 0.1 μg/mL) compared to the control, heart rates for the 20 nm SiO_2_ NPs groups increased with dosage (Figure 2). The differences between the heart rate of the control series and that of high-concentration 20 nm groups (1.0 and 10 μg/mL) were statistically significant. Similarly, the heart rates of the 50 nm groups increased overall, and all but 0.01 μg/mL showed statistically significant differences from the control. 

**Figure 3:**
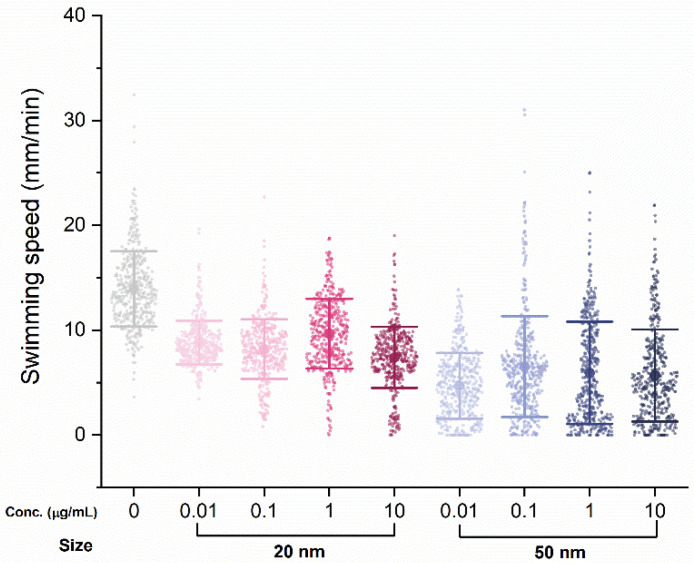
Swimming speed changes upon exposure to SiO_2_ NPs after 48 h. Bars express standard deviations; small dots indicate swimming distance per minute (mm/min); central dot indicates the mean.

Another five individuals were randomly selected from each group to test swimming performance, in which swimming distance was tracked individually, every minute for 80 min. There was a clear decrease in swimming performance with SiO_2_ NPs treatment, with the average swimming distance per minute for the control group (13.2 mm) dropping for both the 20 nm (8.99 mm) and 50 nm (5.52 mm) groups (Figure 3). However, the difference in swimming distance among concentrations within the same nanoparticle size was not significant or dose-dependent. 

### 3.4. Chronic Effects on D. magna Growth

**Figure 4:**
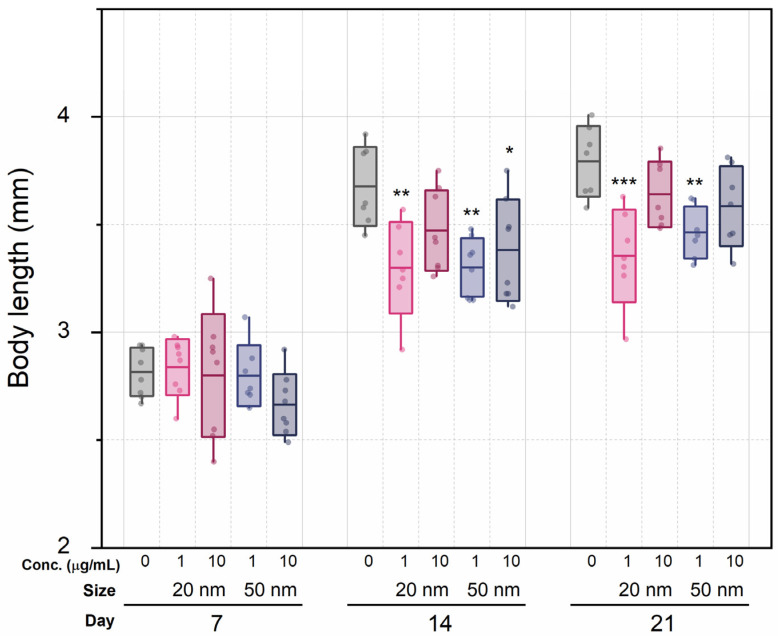
Body length of individuals in each treatment group during 7, 14, or 21 d exposure to SiO_2_ NPs at two concentrations. Boxes express standard deviations; central line indicates the mean. *, ** and *** indicate *p* < 0.05, *p* < 0.01 and *p* < 0.001 relative to the control group, respectively.

We hypothesized that sublethal concentrations of SiO_2_ NPs would induce food assimilation problems, affecting growth. Although dose dependency and nanoparticle size had no effect, the mere presence of SiO_2_ NPs affected the size of mothers (Figure 4) and neonates (Figure S3). The average lengths of the control series were 2.82 mm (7 d), 3.68 mm (14 d), and 3.79 mm (21 d), while the averages of both exposure groups were 2.76 mm (7 d), 3.36 mm (14 d), and 3.51 mm (21 d). At the 7 d mark, there were no statistically significant differences in growth between all groups (one-way ANOVA with Tukey comparisons test). However, at 14 d, body length between the control series and the 1 μg/mL groups showed statistically significant differences (*p* < 0.01 for both sizes), which was maintained at 21 d. The difference in growth increased over time in the low-concentration groups, but in the high-concentration groups, this was not statistically significant. Similarly, neonates from the control group had an average length of 0.949 ± 0.016 mm, as compared with the experimental group lengths of 0.915 ± 0.024 mm (20 nm, 1 μg/mL), 0.901 ± 0.019 mm (20 nm, 10 μg/mL, *p* <0.05), 0.898 ± 0.053 mm (50 nm, 1 μg/mL, *p* < 0.05), and 0.881 ± 0.055 mm (50 nm, 10 μg/mL, *p* < 0.01). 

### 3.5. Accumulation of SiO_2_ NPs in D. magna

**Figure 5:**
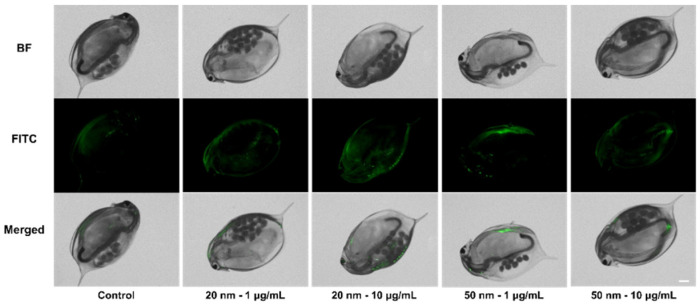
Fluorescence images of *D. manga* exposed to SiO_2_ NPs for 21 d, followed by incubation in fresh media for another 3 d. Scale bar = 500 μM. BF = Bright field.

**Figure 6:**
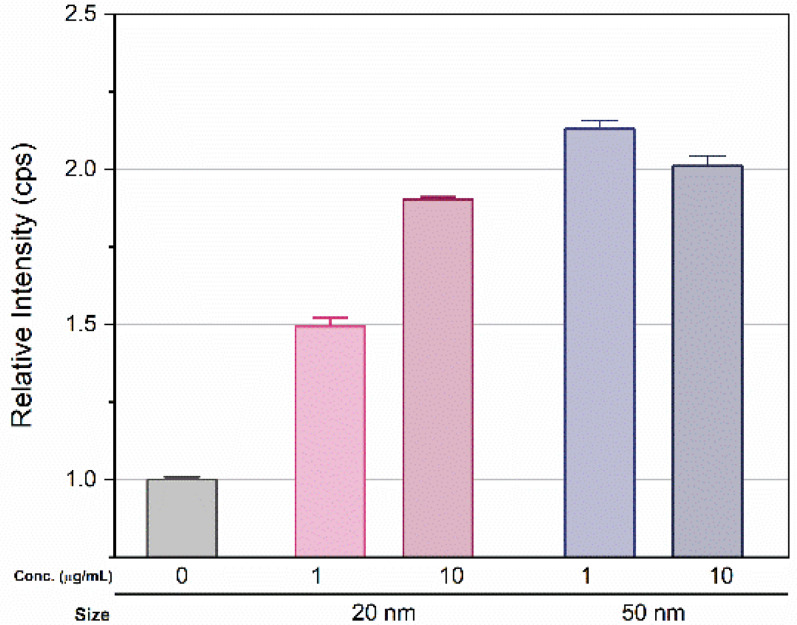
Relative intensity of Si in *D. magna* (measured by ICP-MS) exposed to 1 and 10 μg/mL concentrations of 20 and 50 nm SiO_2_ NPs for 27 d, including washing in ISO medium. Error bar indicate standard deviation.

After 14 d exposure, green fluorescence was observed in *D. magna*. The brightness increased proportionally with the applied concentration, but no remarkable change was observed by exposure time (Figure S4). *D. magna* were then transferred to an untreated condition identical to that of the control series, followed by 3 d incubation. Interestingly, fluorescence remained in *D. magna* exposed to SiO_2_ NPs (Figure 5). Next, the amount of SiO_2_ uptake in *D. magna* was measured using ICP-MS. After the visualization experiment, *D. magna* were transferred to fresh media and incubated for another 3 d to minimize the influence of SiO_2_ NPs. The measured relative amount of silicon ions in the exposure groups showed a > 1.5-fold increase compared to those of the control series (Figure 6). In the 20 nm SiO_2_ NPs, the relative intensity seemed to depend on the concentration, while the 10 μg/mL 50 nm Si nanoparticles had a lower intensity than those at 1 μg/mL, and the relative intensities showed negligible differences. 

## 4. Discussion

### 4.1. Acute and Chronic Effects on D. magna Mortality, Reproduction, and Growth

The toxicity of SiO_2_ NPs can change significantly based on size, shape, and functionalization. Vicentini et al. [14] reported the acute toxicity of SiO_2_ NPs by comparing two morphologies, calculating 48 h half-maximal effective concentrations (EC_50_) of 4.9 mg/mL (tubular) and 2.2 mg/mL (spherical). A comparison between amine-functionalized and 50 nm spherical SiO_2_ NPs showed that the acute toxicity of the SiO_2_ NPs was 2.2 mg/mL (EC_50_) [13]. According to Clement et al. [15], the toxicity of amorphous 14 nm SiO_2_ NPs at 72 h was 29.7 mg/L (EC_50_), and these nanoparticles caused morphological alterations and cellular-level changes including mitochondrial deformation in tissues and deformed nuclei in *D. magna* eggs. It appears that a lack of direct lethality related to SiO_2_ NPs should not discourage the investigation of sublethal and chronic effects. Our acute toxicity tests were carried out under limited concentrations (<100 μg/mL) at 48 h, according to OECD test guideline 202. Spherical SiO_2_ NPs of both sizes would not affect immobilization on *D. magna*, as the acute toxicity of SiO_2_ NPs is lower for spherical and unfunctionalized conditions. In the duplication test, only eight daphnids showed mortality over a total of 660 individuals. Considering a few mortalities from the control series, the effect of SiO_2_ NPs on acute toxicity was negligible under the applied conditions. 

Two studies have shown that SiO_2_ NPs lead to an increase in *D. magna* reproduction: this rose by 18% with a slurry of colloidal SiO_2_ NPs, while commercially available 7 and 10 nm SiO_2_ particles slightly increased reproduction [38]. On the other hand, Vicentini et al. [14] reported that reproduction decreased significantly upon exposure to concentrations of 50 mg/L 80 nm SiO_2_ particles. These conflicting results can be used to elucidate size and surface differences [13]. In our chronic toxicity test, SiO_2_ NPs in the 20 nm and 10 μg/mL groups gave rise to slightly higher reproduction compared to the control series. Nevertheless, the average number of offspring in all groups did not show statistically significant differences, demonstrating that the reproduction of *D. magna* was not affected by SiO_2_ NPs under the applied conditions. However, a growth analysis showed that both SiO_2_ NPs sizes caused growth retardation that was significant relative to the control, beginning from day 14 of exposure until study termination (day 21). Previous studies exploring this particular feature are rare, though Lee et al. [38] indicated no significant change in size with exposure to 7 and 10 nm particles. A comparison by Karimi et al. [39] of different silica oxides (colloidal and fumed silica) reported that the latter (120–140 nm particles) did not result in size changes with acute exposure, but the former (50–60 nm particles) increased the size of *D. magna* by up to 24%; on the other hand, chronic exposure (21 d) to both compounds increased the size by 10%. These changes were attributed to hormetic adaptation, and the authors concluded that exposure to silica oxides did not have a significant adverse effect on *D. magna*. Similar results (increased size with long-term exposure) and an almost identical conclusion were described by Lillicrap et al. [40], who investigated the effects of low-grade and high-grade silica fumes on *D. magna*.

### 4.2. Effects of SiO_2_ NPs on D. magna Swimming Performance and Heart Rate

Swimming performance has been used for the assessment of low-toxicity materials in cases where mortality and immobilization are difficult to identify. Observations of *D. magna* heart rate are also recognized as a sublethal endpoint for toxicity screening. Decreased speed or other adverse effects on swimming have been reported in previous studies exploring titanium, nano-C_60_, copper, and other metals or compounds [41,42,43,44]. Although we did not observe dose dependency, swimming speed decreased significantly within our exposure groups and the 50 nm groups showed slower swimming speeds than the 20 nm groups. Heart rate increased significantly in the 20 nm (1 and 10 µg/mL) and 50 nm (0.1, 1, and 10 µg/mL) groups relative to control. These changes are similar to those reported in prior studies using heartbeat as an endpoint for exploring fullerenes (nano-C60), graphene oxide, titanium oxide, and copper-based nanoparticles [30,41,42,45]. This may be caused by the absorption of SiO_2_ NPs because the toxic effect of nanoparticles on signal pathways has not been clearly demonstrated [44,46]. When considered alongside physiological/behavioral and growth changes, this may suggest that SiO_2_ NPs led to the obstruction of food assimilation and the physical restriction of movement [44,46,47,48,49]. 

### 4.3. Accumulation of SiO_2_ NPs in D. magna

Small zooplanktonic organisms are able to absorb micro- and nano- particles as food because they are filter feeders [49]. Although we found no dose-dependent fluorescence brightness, relatively strong fluorescence from exposed *D. magna* showed that small particles could be absorbed and accumulated through exposure. Given that the fluorescence brightness did not change significantly for over 21 d, the accumulation seemed to reach saturation at some point. The quantitative amount of silicon ion uptake was not precisely measured because of the fairly high intensity in the control series, possibly due to uptake from the cultural media. For this reason, we expressed the amount of silicon ions by normalizing the intensity from the control series. The measured relative amount of exposure samples showed more than a 1.5-fold increase over that of the control (Figure 6). The relative intensity of the silicon ions was affected by the size rather than the concentration, and increased with size. For the 20 nm particles, the relative intensity seemed to depend on the concentration, showing a difference of approximately 35% between the two concentrations. In contrast, 10 μg/mL of 50 nm particles had a lower intensity than 1 μg/mL, and the relative intensities had negligible differences. As a result, there was no significant consistency between nanoparticle uptake in *D. magna* and applied Si concentration due to saturation, but uptake and accumulation was clearly observed.

### 4.4. Conclusions

As expected from previous research, spherical SiO_2_ NPs did not lead to acute or severe chronic effects on *D. magna*. However, physiological and behavioral changes were observed in adults and neonates. The heart rate and swimming performance, which are strongly related to the health and survival of *D. magna*, were affected by SiO_2_ NPs in the environment. The length of *D. magna* mothers and neonates also decreased compared to that of the control group. Such growth factors could have been affected by food assimilation alterations by the accumulation of SiO_2_ NPs in the body. Accumulation trends were validated by ICP-MS and fluorescent imaging. Although the results did not demonstrate mortality, immobility, or reproductive adverse effects, as swimming performance and heart rate are regarded as reliable proxies for identifying toxicity in *D. magna*, these results appear to support the hypothesis that SiO_2_ NPs have toxic effects on this species. Physiological and behavioral endpoint experiments may not be sufficient to elucidate the toxicity of SiO_2_ NPs from an adverse outcome pathway or mechanistic point of view. However, for a better understanding of the effects of low-toxicity nanoparticles under sublethal concentrations, these endpoint tests provide an overview of sublethal toxicity.

## Supplementary Materials

The following are available online at https://www.mdpi.com/article/10.3390/mi12091105/s1, Figure S1: Survival (%) of *D. magna* upon exposure to SiO_2_ NPs after 48 h, Figure S2: The number of total neonates reproduced during 21 days, Figure S3: Body length of neonates for each treatment group, Figure S4: Fluorescence images of *D. magna*, Figure S5: The calibration curve of silicon ion measured with ICP-MS.
