# Supplementary material for: Physiological and Behavioral Effects of SiO2 Nanoparticle Ingestion on Daphnia magna
Source: Micromachines (Basel). 2021 Sep 14;12(9):1105. doi: 10.3390/mi12091105 (PMC8472362; doi:10.3390/mi12091105)
Supplement: Supplementary file 1 [file micromachines-12-01105-s001.zip › micromachines-1366326-supplementary.pdf]

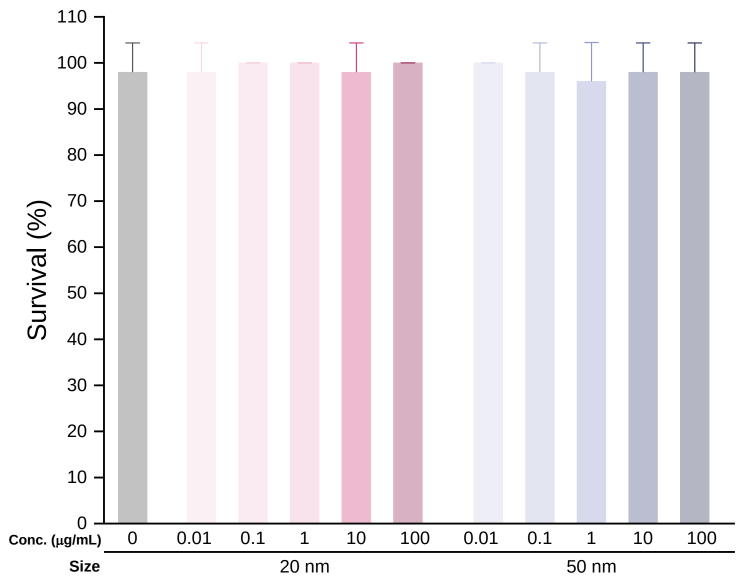

Figure S1. Survival (%) of *D. magna* upon exposure to SiO_2 NPs after 48 h. Neonates were exposure to SiO_2 NPs of various concentrations (0.01, 0.1, 1, 10, 100 µg/mL). Bar indicates the mean. Error bar express standard deviations.

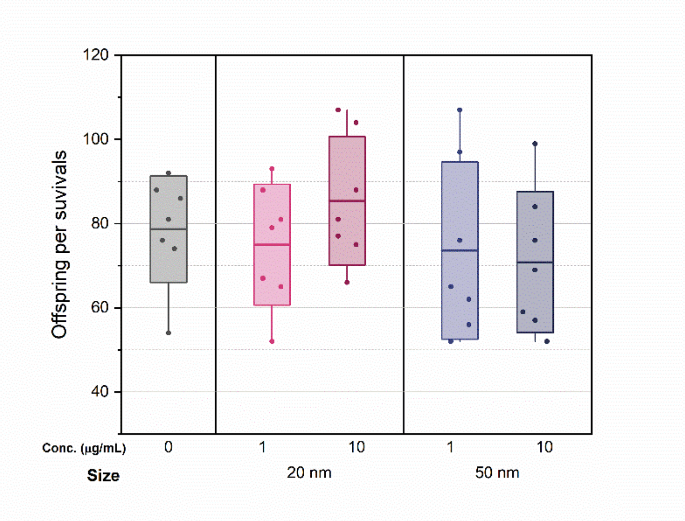

Figure S2. The number of total neonates reproduced during 21 days. The mothers were exposed to SiO_2 NPs (20 and 50 nm) of two concentrations (1, and 10 µg/mL). Boxes express standard deviations; central line indicates the mean.

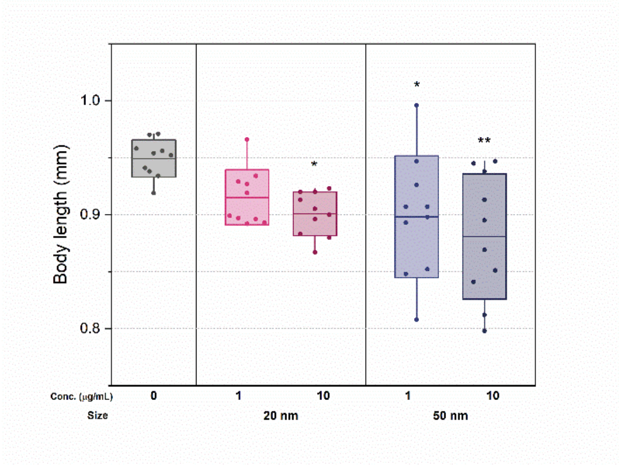

Figure S3. Body length of neonates for each treatment group. The 3rd brood of ten neonates was randomly pooled. Boxes express standard deviations; central line indicates the mean. * and ** indicate $p < 0.05$ and $p < 0.01$ relative to the control group, respectively.

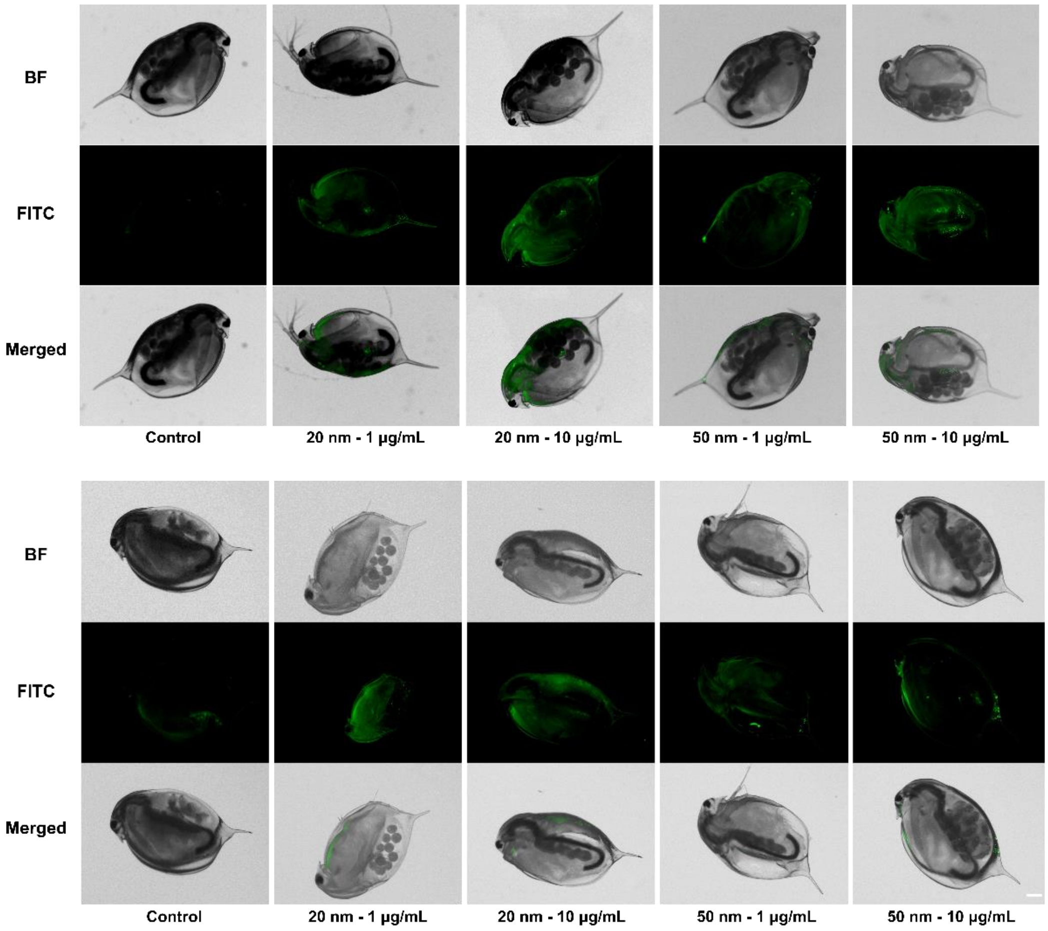

Figure S4. Fluorescence images of *D. Magna*. Daphnids were exposed to SiO_2 NPs for 14 (above) and 21 (below) days. Scale bar = 500 μM. BF = Bright field.

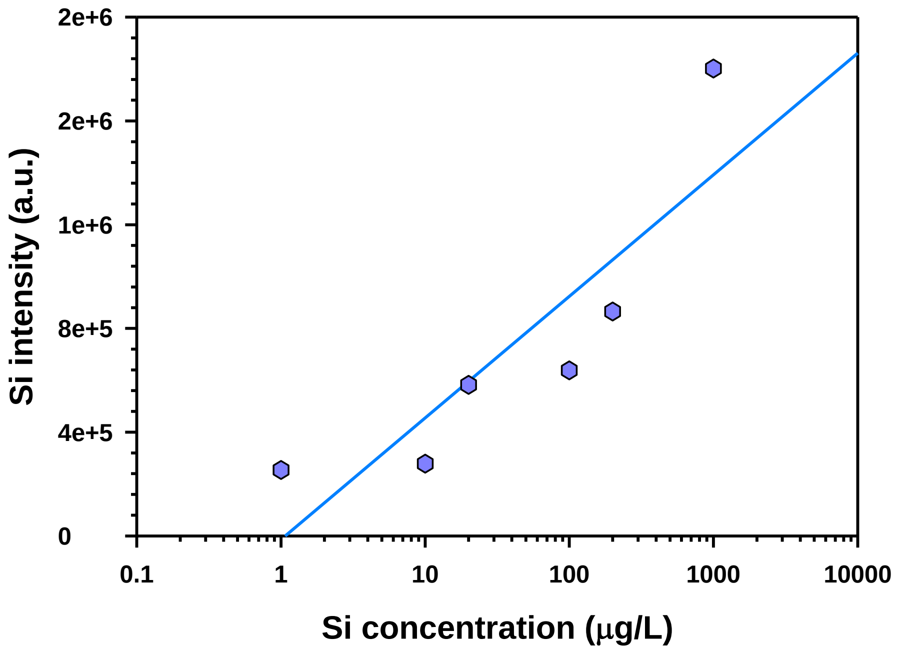

Figure S5. The calibration curve of Si measured with ICP-MS. Standard Si concentration was plotted in log scale and R-square value is 0.9367. This value has an error of ±0.008 as the standard solution has relatively broad error concentration as 997 ±4 mg/L.
